# Radiation-induced cavernoma in pediatric CNS tumors: a systematic review and treatment paradigm

**DOI:** 10.1007/s00381-024-06543-0

**Published:** 2024-07-19

**Authors:** Adam Vacek, Chandrasekaran Kaliaperumal

**Affiliations:** 1 The University of Edinburgh Medical School, Edinburgh, UK; 2 Department of Neurosurgery, Aberdeen Royal Infirmary, Aberdeen, UK; 3 Department of Clinical Neuroscience, Royal Infirmary of Edinburgh, Edinburgh, UK

**Keywords:** Pediatric, Radiation, Induced, Cavernous, Malformation, Cavernoma

## Abstract

**Purpose:**

This retrospective systematic literature review aimed to summarize available data regarding epidemiology, etiology, presentation, investigations, differentials, treatment, prevention, monitoring, complications, and prognosis for radiation-induced cavernous malformations (RICMs) in pediatric patients.

**Methodology:**

Review conducted per PRISMA guidelines. Google Scholar, PubMed, Trip Medical Database, and Cochrane Library searched utilizing a keyphrase, articles filtered per inclusion/exclusion criteria, duplicates excluded. Based on criteria, 25 articles identified, 7 further excluded from the systematic data but included in discussion (5 × insufficient data, 2 × other systematic reviews).

**Results:**

Many studies did not contain all explored data. 2487 patients reviewed, 325 later found to have RICM (143 male, 92 female). Mean age at irradiation 7.6 years (range 1.5–19). Mean total radiation dose 56 Gy (12–112). Most common indications for radiation—medulloblastoma 133x, astrocytoma 23x, ependymoma 21x, germinoma 19x. Mean age at RICM diagnosis 18 years (3.6–57). Mean latency to RICM 9.9 years (0.25–41). Most common anatomic locations—temporal 36, frontal 36, parietal 13, basal ganglia 16, infratentorial 20. Clinical presentation—incidental 270, seizures 19, headache 11, focal neurological deficit 7, other 13. 264 patients observed, 34 undergone surgery. RICM bled in 28 patients. Mean follow-up 11.7 years (0.5–50.3). Prognostic reporting highly variable.

**Conclusions:**

From our data, pediatric RICMs appear to display slight male predominance, present about 10 years after initial irradiation in late teen years, and present incidentally in majority of cases. They are mostly operated on when they bleed, with incidental lesions mostly being observed over time. Further prospective detailed studies needed to draw stronger conclusions.

## Introduction

Recent oncological developments have led to improvements in prognosis for childhood cancer patients. In 2005, there were an estimated 330,000 survivors of childhood cancer in the United States, with 5-year survivorship rates increasing to > 80% nowadays [1]. Some of these recent advances include more advanced chemotherapy regimens and radiation treatment plans and techniques [1, 2]. While radiotherapy was previously mainly utilized in treatments of leukemias and lymphomas, its usage has expanded to treating many central nervous system tumors, where it has been shown to improve survival rates [1, 3].

However, these treatments do not come without risks. Many survivors of childhood cancers are left with long-term sequelae of their treatments. A longitudinal cohort of almost 10,400 participants found that over 60% reported at least one chronic health condition and almost 30% described severe sequelae by a mean age of 26.6 years [1]. Radiation-induced cavernous malformations (RICMs) represent one potential long-term complication of brain irradiation in pediatric patients [1, 3]. RICMs are reported as the most common magnetic resonance imaging (MRI) abnormality in long-term survivors [4], although cumulative incidence rates vary significantly in published literature [4]. The mechanism by which radiotherapy influences the formation of RICMs is unknown, as are aspects of the pathophysiology of cavernous malformations themselves [4, 5]. Whether there are any particular risk factors predisposing patients to the development of RICMs is also unclear [4].

RICMs are also often found incidentally in follow-up surveillance studies, though no clear guidelines on RICM follow-up monitoring of childhood cancer survivors exist [6]. It does not help that these lesions often take a long time to develop, with latency periods often measured in years [7]. These lesions are often simply followed up without an intervention, though they can cause seizures, headaches, and various other symptoms, and can bleed, often requiring neurosurgical intervention [6]. However, there is a lack of unified consensus surrounding many aspects most effective treatment paradigms and neurosurgical care for pediatric patients with RICMs.

### Objectives

This retrospective systematic literature review aimed to summarize available data regarding epidemiology, etiology, clinical presentation, investigations, differentials, treatment paradigms, prevention, monitoring, complications, and prognosis for RICMs in pediatric patients, helping to form a consensus regarding some of the unclear aspects surrounding this pathological phenomenon.

## Materials and methods

### Search strategy (Fig. 1)

**Fig. 1:**
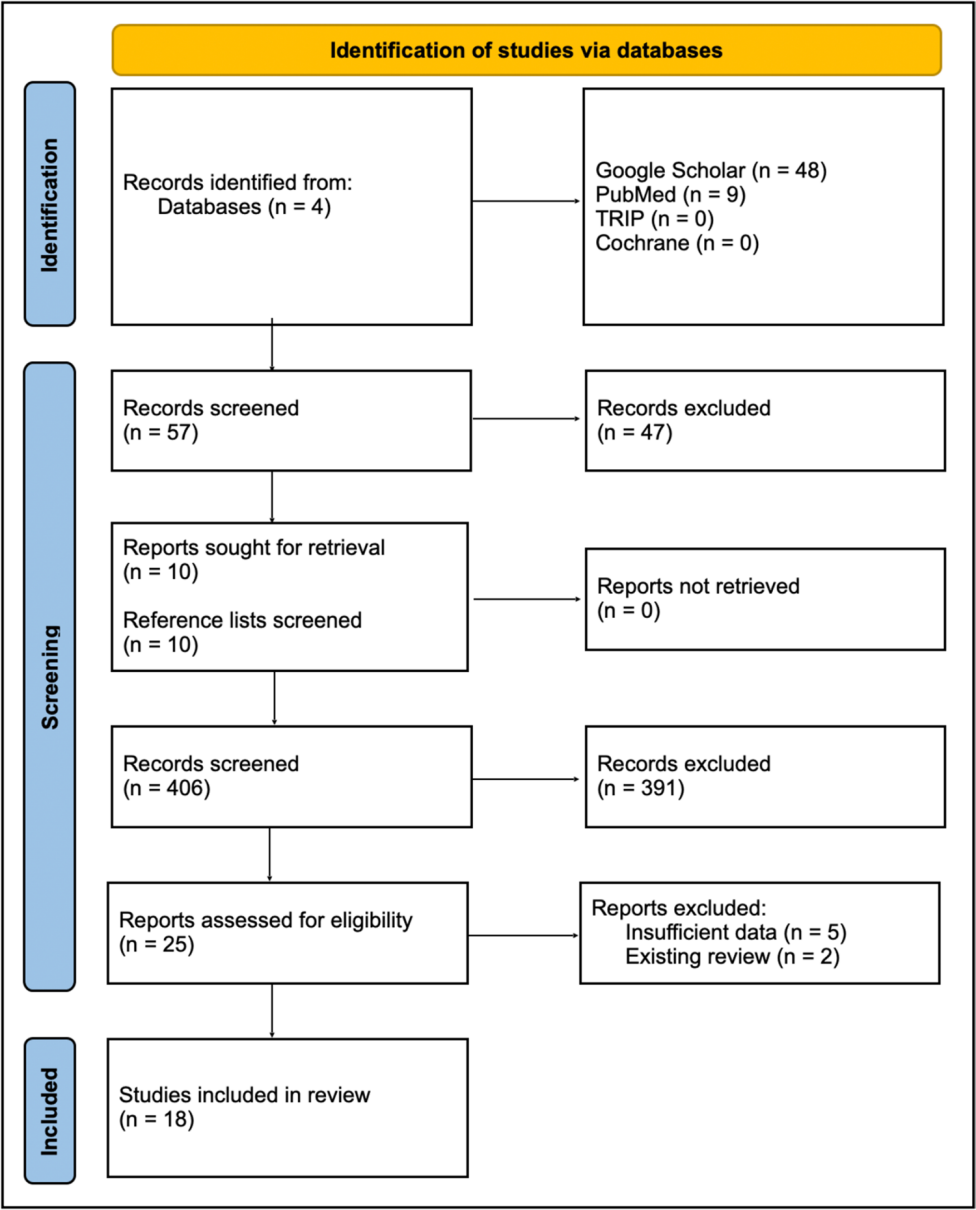
Search strategy breakdown

The study was conducted according to the PRISMA guidelines [8] (PROSPERO registration CRD42023481843). Google Scholar, Trip Medical Database, Cochrane Library, and PubMed were searched in March 2024 for all available literature utilizing the following key phrases: “cerebral” [all fields] AND “children” [all fields] OR “paediatric” [all fields] OR “paediatric” [all fields] AND “cavernous malformations” [all fields] OR “cavernoma” [all fields] AND “radiation induced” [all fields] OR “radiation-induced” [all fields]. All articles written in English were included; no articles were excluded based on study type, publication date, or status. Articles were screened by a single researcher to ensure relevance to the searched key phrase and ensure they relate to radiation-induced cavernomas and pediatric patients at the time of irradiation for CNS tumors. First, articles were screened based on title and abstract. Subsequently, full text of included articles was screened, and reference lists of included articles were searched in the same way. All duplicates were excluded. In total, 463 papers were screened (including duplicates), from which 25 papers were included.

### Study records and data items

Reported parameters include study parameters, number of patients reviewed, number of RICM patients, sex, age at radiation, total radiation dose, indication for irradiation, age at RICM diagnosis, site of RICM, latency to RICM post radiation, clinical presentation, treatment approach, complications of RICM, follow-up time, and prognosis. Additional explored and discussed parameters include epidemiology, etiology and pathophysiology, investigation modalities, differentials, prevention, monitoring, and study bias.

### Data synthesis

For data synthesis, there was no minimum number of studies required for data to be presented for a particular category. Editorials and case reports were included, literature/systematic reviews were excluded, and primary data sources were used. Due to the limited size of each individual patient series, individual patient data analysis (IPDA) and analysis of the raw data were conducted in favor of general meta-analysis. For quantitative data, means and ranges were calculated as they were the most frequently available metrics in the included literature. Qualitative data were synthesized via a narrative synthesis into a presentable data segment for the academic output. Where data was missing or it was impossible to extract data relating only to patient groups of interest, these were marked as “unknown” and were not included in data synthesis for the relevant category. Where studies reported metrics for the entire studied group and not only RICM patients, we attempted to extract data pertaining only to the RICM patients, removed any data entries where this was not possible, and marked these as “unknown”.

### Bias and meta-bias

For assessing risk of bias, and meta-bias, the Robvis tool was used, with data recorded in the RoB2.0 format by a single researcher [9]. The GRADE framework was utilized to categorize the quality of data [10].

## Results

Characteristics of the 25 included studies can be found in Table 1. Five studies were excluded from the IPDA as they did not provide sufficient information about the RICM cohort of interest [1, 3, 6, 11, 12]. Same applies to the 2 existing literature reviews, where we instead searched for the original data sources [2, 13]. Many studies did not contain information on all explored data categories.

**Table 1:** Included studies’ characteristics

Study	Year	Study characteristics	Number of patients reviewed	Number of patients with cavernoma
Chang, S. [14]	1998	Case report	Unknown	1
Duhem, R. [5]	2005	Case series	419	9
Mukae, N. [15]	2019	Case report	Unknown	1
Lew, S. M. [16]	2006	Retrospective review	59	18
Liby, P. [17]	2017	Case report	Unknown	1
Campbell, B. A. [4]	2021	Retrospective review	467	113
Larson, J. J. [18]	1998	Case series	Unknown	6
Burn, S. [19]	2007	Retrospective review	379	10
Strenger, V. [20]	2008	Retrospective review	171	8
Passos, J. [21]	2015	Case series	121	19
Passos, J. [22]	2017	Retrospective review	132	Unknown
Di Giannatale, A. [23]	2014	Retrospective review	108	34
Pozzati, E. [24]	1996	Case series	Unknown	5
Baumgartner, J. [25]	2003	Case series	Unknown	3
Trybula, S. [26]	2021	Retrospective review	79	68
Jain, R. [27]	2005	Retrospective review	Unknown	5
Kamide, T. [28]	2010	Case report	Unknown	1
Vinchon, M. [29]	2011	Retrospective review	552	23

The total number of reviewed patients was 2487, with 325 previously pediatric irradiated patients developing RICM later in life, 143 males and 92 females (male to female ratio of roughly 3:2). Mean age at radiation was 7.6 years (range 1.5–19); mean total radiation dose was 56 Gy (range 12–112). The most common indications for irradiation included medulloblastoma 133x (40.9%), astrocytoma 23x (7.1%), ependymoma 21x (6.5%), and germinoma 19x (5.8%). Cavernoma, craniopharyngioma, dysgerminoma, ganglioglioma, glioma, pituitary adenoma, and rhabdomyosarcoma each occurred once in the cohort. There were also 4 cases of CNS lymphoma and 4 cases of “other CNS malignancies”. Notably, a few studies also included several patients with leukemia (total of 27) and “other non-CNS malignancies”; however, due to insufficient depth of provided data, we were unable to remove these from the IPDA.

The mean age at RICM diagnosis was 18 years (range 3.6–57), with a mean latency post-radiation of 9.6 years (range 0.25–41). The anatomical location of RICMs is as follows: temporal lobe 36 patients (11%), frontal lobe 36x (11%), parietal lobe 13x (4%), basal ganglia 16x (4.9%), cerebellum 11x (3.4%), “infratentorial” 7x (2.2%), occipital lobe 5x (1.5%), limbic lobe 5x (1.5%), brainstem 1x, foramen of Monro 1 patient. RICM was diagnosed incidentally in 270 patients (83%), 19 presented with seizures (5.8%), 11 with headache (3.4%), 7 × focal neurological deficit (2.2%), 5 × nausea and vomiting (1.5%), 3 × ataxia (0.9%), 3 × hearing loss (0.9%), 1 × vision disturbance, and 1 with loss of consciousness.

Two hundred sixty-four patients were simply observed (81.2%), with 34 patients undergoing surgery (10.5%). The cavernoma bled on presentation in 28 patients (8.6%). Other indications for surgery included new-onset seizures, increased seizure frequency, new neurological deficits, and substantial interval growth. The mean follow-up duration was 11.7 years (range 0.5–50.3), although only 6 studies reported any data on follow-up for the RICM cohort. While reported prognostic data vary widely and are not reported for the vast majority of patients, a simple summary can be made. With observation, 2 patients improved, 28 remained stable, and 7 worsened. With surgery, 17 patients improved, and 7 patients worsened. The prognosis is unknown for 269 patients (82.8%).

**Fig. 2:**
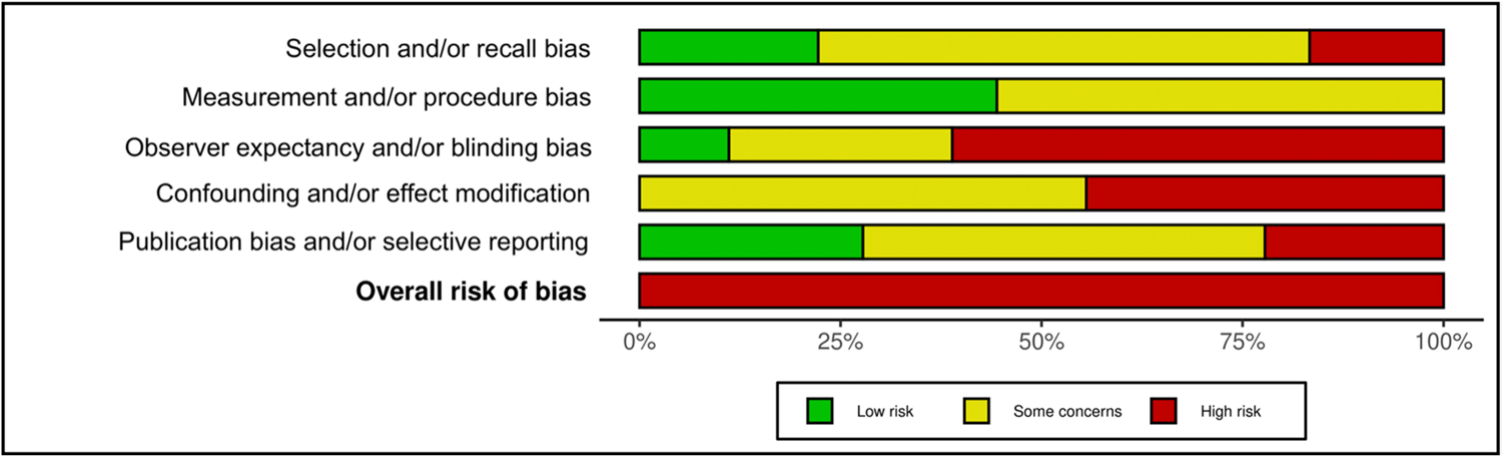
Robvis bias analysis

**Fig. 3:**
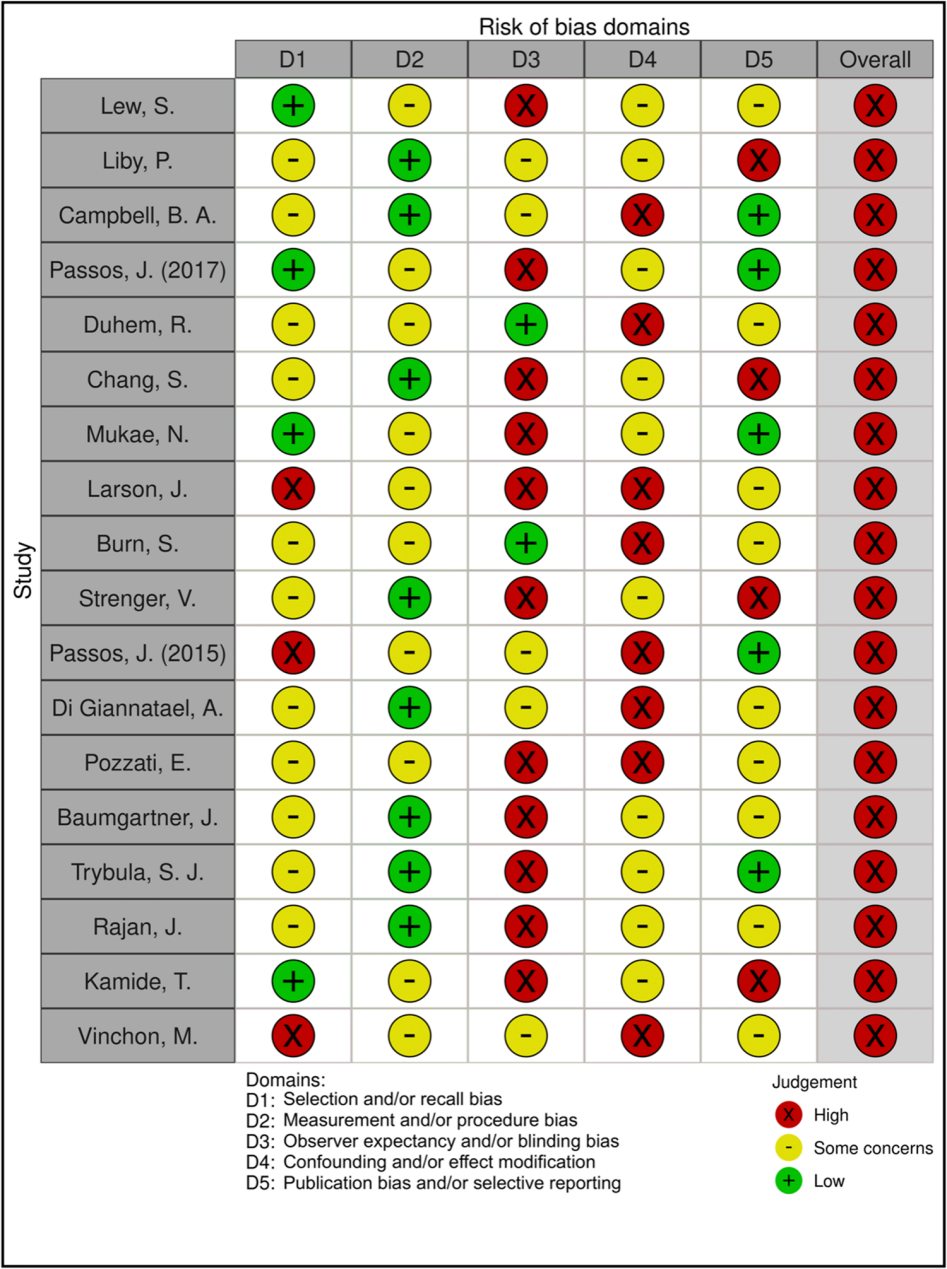
Robvis bias analysis

Data from Robvis bias analysis are summarized in Figs. 2 and 3. The GRADE quality of data was estimated to be at moderate to low levels, pending the data category. This is mainly due to the small sample sizes, lack of reported data for several categories, and inherent biases associated with case reports and retrospective studies, all of which decrease the overall quality of the pooled data.

## Discussion

In summary, our data review includes 325 previously irradiated pediatric patients who developed RICM later in life, with a male-to-female ratio of 3:2 and mean age at the radiation of 7.6 years, with a mean radiation dose of 56 Gy. The most common indication for irradiation was medulloblastoma (40.9%). The mean age at RICM diagnosis was 18 years, with a mean post-radiation latency period of 9.6 years. RICMs occurred most in frontal and temporal lobes in these patients (combined 22%). The majority (83%) of patients presented asymptomatically with incidental findings of RICM, and 28 patients (8.6%) experienced cavernoma bleeding on presentation. 81.2% of patients were simply observed, and 34 patients (10.5%) underwent surgery. Prognostic data is largely missing, with prognosis unknown for 82.8% of patients. While the overall quality of the data is low, with high risks of bias, owing to the majority of the included literature consisting of case reports or small case series, the data nonetheless presents several intriguing discussion points.

The natural incidence of cavernomas in the general population varies in the literature (0.02–0.53%), with the most frequently quoted number being 0.5% [7, 19, 30]. As radiation-induced cavernomas are reported mostly as sporadic case reports, their incidence is difficult to calculate. While the cohort of studies we reviewed includes 2487 patients, with 325 later developing RICM resulting in an “incidence” of 13.1%, or 26 × larger compared to the general population statistics, it is unclear from the literature how many of the 2487 patients actually received prior radiation treatment for any form of CNS pathology. The cumulative incidence in published literature varies between 3 and 43% at 10 years post-radiation [4, 7, 20]. This variability has been attributed to the differences in evaluated population, screening patterns, and sensitivities of imaging modalities used [4]. Other reports include sixfold increases in the risk of developing cavernoma in irradiated patients compared to the general population [7]. This could also help explain the higher incidence of supratentorial RICMs, as medulloblastoma patients receive lower dose to the supratentorial compartment, compared to the dose given to the infratentorial posterior fossa (generally given as a radiation boost). Though we may be aware of the cumulative radiation dose responsible for radiation-induced CNS malignant tumors[31], there remains uncertainty about the radiation dose responsible for the induction of cavernous malformations.

Some cavernous malformations are considered congenital, especially in familial forms and in young infants [14, 24]. Others propose that the majority of cavernous malformations in adults and children are considered acquired [14, 25]. Another very different hypothesis postulates that inert cavernomas were already present in some patients later found to have RICM and the radiation treatment simply included their growth [24]. The etiology and pathophysiology for both naturally-occurring and radiation-induced cavernomas remain unclear, although several theories have been postulated. The effects of radiation on vasculature include induction of capillary proliferation, fibrinoid necrosis of vascular walls, endothelial hyperplasia, hyalinization, and luminal stenosis [5, 12, 14, 27]. Additionally, it is thought that microhemorrhages from fragile, radiation-affected vessels may trigger angiogenic factors, contributing to vascular malformations [14]. Perivascular lymphocytes, with potential anticoagulation properties, may also contribute to RICM growth [14]. It has also been postulated that venous restrictive disease occurring due to radiation change, resulting in impaired venous flow and increased venous pressure, can contribute to cavernoma formation [16]. The radiation dose–response relationship is being explored, but RICM formation has been described at both high and low radiation doses. One theory postulates that low doses may be more conducive to RICM formation, as high doses may cause extensive cellular apoptosis, preventing RICM formation [7]. This is thought to explain some findings of RICMs often occurring at the edge of the radiation treatment field, in addition to the ones occurring in the center of the field [7].

In regard to growth factors, high expression of vascular endothelial growth factor (VEGF), basic fibroblast growth factor (bFGF), and transforming growth factor (TGF) have been observed in irradiated cells and are thought to play a role in RICM pathogenesis [5, 20]. Some of these angiogenic factors are also expressed at higher levels in children, which could help explain why children could be more susceptible to RICM development than adults [7]. Furthermore, genetic predisposition may also play a role. One theory suggests a genetic predisposition from somatic mutations similar to familial cavernomatosis, with direct DNA damage from radiation acting as a “second hit” [7]. Germinal mutations and structural protein immaturity have also been postulated to play a role as defects contributing to cavernomas [5].

Demographic information and focus on risk factors vary between the studies. While some have reported slightly higher male prevalence [7, 23], others report slightly higher female preponderance [5, 12]. Our results pooling together all available data on 325 patients show a male-to-female ratio of roughly 3:2 (143 males, 92 females), which would indicate a slight male prevalence for RICMs. While age sensitivity is still a controversial topic, suggestions of potential effects are arising. It has been suggested that children irradiated at younger ages (< 10 years) are at higher risk of developing RICMs and also show shorter latency intervals for RICM development [4]. Our data would support this notion, with a mean age at irradiation of 7.6 years. While the above-described potency of low-dose radiation is still being explored, other studies have described that higher radiation doses are associated with shorter latency intervals and occurrence of RICMs [12, 30], some also suggesting a dose–response relationship, with higher doses correlating to higher RICM frequency [30]. Other studies, however, report no significant relationships between radiation dose and RICM development [20]. The high variability of reporting quality of the relevant data prevents us from drawing any impactful conclusions from our pooled data. Few studies looking at the effects of adjunctive chemotherapy to radiation showed no significant relationship [21], although one study looking at RICMs in leukemia patients (excluded from our analysis as not related to CNS tumors) has described methotrexate treatment followed by radiotherapy showing higher incidence and shorter latency for RICMs compared to radiotherapy alone [23]. Only a few studies explored the effects of the underlying disease, with one finding that underlying disease (brain tumor vs. other solid tumors vs. leukemia vs. TBI) showed no significant influence on the development of RICM [20]. A second study evaluating patients undergoing hematopoietic stem cell transplants and radiation (also excluded from our analysis due to a lack of focus on CNS tumors) showed that patients with malignant diseases showed a significantly higher frequency of RICMs and a significantly higher probability of developing RICMs, compared to those with nonmalignant diseases [30]. Lastly, some have suggested that certain parts of the brain may be more susceptible to radiation than other, with a majority of cavernomas presenting in supratentorial areas, despite frequent irradiation to infratentorial areas for medulloblastomas [1, 6, 23, 30]. Our data would support this notion, with 111 patients (34.2%) in our review presenting with supratentorial RICM, compared to 20 patients (6.2%) presenting with infratentorial RICM, all while the most common reason for irradiation was a medulloblastoma (40.9%), which in the vast majority of pediatric cases occur infratentorially. However, without detailed data for each patient demonstrating the location of their primary pathology, the location of irradiation, and the location of subsequent RICMs, it is difficult to draw definitive conclusions. It is also important to point out the supratentorial brain volume is higher compared to the infratentorial volume, providing more opportunity for RICMs to arise. Additionally, the increased incidence in medulloblastoma patients could also in some extent be attributed to different radiation protocols. Patients with medulloblastoma receive radiation to the whole neuroaxis (craniospinal irradiation), while pediatric patients with other primary pathologies (e.g., ependymoma or germinoma) may only receive craniospinal irradiation if there is evidence of leptomeningeal dissemination.

In agreement with our data (83% asymptomatic at presentation), researchers agree that the vast majority of pediatric RICM patients present asymptomatically [4, 7]. The precise number varies, from studies reporting 50% asymptomatic at presentation [6] to symptom-free period of 5 years, 10 years, and 15 years post-diagnosis of 98%, 96%, and 91% respectively [4]. Two presentations of concern include seizures and hemorrhage. In our data, we found 8.6% of patients presented with hemorrhage and 5.8% presented with seizures. Available literature does not provide much further detail; one study mentions that about half of pediatric patients with RICM who present with symptoms can experience seizures [6]. Hemorrhage is a much more deeply explored area. In the pediatric population, the rate of hemorrhage for non-radiation-induced cavernomas is reported around 0.25–3%, with many studies quoting numbers below the 1% range [4, 7, 28, 32]. In comparison, pediatric RICM hemorrhage rate is reported to be much higher, ranging from roughly 4% to roughly 36% [5, 7, 23, 32]. Research into factors that could increase risk of cavernoma hemorrhage in pediatric patients have not discovered any significant association with age, sex, cavernoma multiplicity, or family history [6]. However, brainstem location, lesion size, presence of perilesional edema, and hemorrhagic clustering (repeated hemorrhage), were found to be significant factors [6].

While CT scans provide a readily available imaging technique to identify hemorrhage secondary to RICMs, they are less sensitive in identifying inert RICMs. The development and availability of MRI have increased the identification of these lesions, with MRI being the imaging modality of choice [6]. Many specialized forms of MRI have been explored for RICM investigation and monitoring, including susceptibility-weighted imaging, diffusion-weighted imaging, contrast MRI, and diffusion tensor imaging[4, 6, 12]; however, no conclusive data or recommendations can be drawn from the available information. In regard to lesion locations, similarly to our data, other studies have mentioned RICM preference for supratentorial regions [1, 6, 23, 30]. Additionally, research shows that RICMs not only develop in the primary radiation site but often also at the margins of the radiation field or at distant sites from the primary radiation site [7, 23]. Of note, RICMs can sometimes be difficult to interpret on imaging, particularly in patients with a history of neoplasia, where the possibility of a new tumor with intratumoral hemorrhage must be considered in contrast to an RICM [6].

Monitoring for RICMs and follow-up for pediatric cancer patients is a highly debated field. While the mean follow-up for our pooled cohort was 11.7 years, only 6 studies reported their follow-up time, and the range was highly variable (0.5–50.3). The current literature correctly argues that the clinical significance of imaging-detected asymptomatic RICMs remains uncertain [4]. While there is no consensus on the precise length or frequency of follow-up, almost all studies recommend some degree of monitoring for these patients. There are obvious benefits to detecting cavernomas early, so they can be initially monitored for any changes or risk of bleeding in the shorter term; however, the long-term frequency of follow-up is unclear. It is correctly noted that the benefits of regular screening must be weighed against the economic and psychological costs to patients [4]. At the moment, routine screening for RICM in long-term childhood CNS cancer survivors is not universally recommended [4]. Some studies recommend MRI screening every two years for the first 18 years, and every 5 years thereafter, based on their data of hemorrhage incidence [4]. Our pooled data show a mean latency post-radiation to RICM diagnosis of 9.6 years, demonstrating that if regular screening was to be recommended, it would likely need to take place over several decades for patients. As for monitoring existing diagnosed RICMs, some suggest that small incidental RICMs require less frequent follow-up as they are less likely to bleed [6]. For hemorrhagic RICMs, studies suggest a more frequent initial follow-up of 3–6 months, followed by annual follow-up thereafter [6].

The management rationale changes based on individual patients. In general, three management pathways are described in the literature—observation, surgical resection, and radiosurgery. The decision-making factors that should be included are the lesion location, symptomatic burden, history of hemorrhage, risk-to-benefit ratio of particular intervention, and patient age and health [6, 7, 23]. As the natural history of RICMs is often benign, with low annual hemorrhage rates, especially in cases of small asymptomatic cavernomas, surgery often carries higher risks than benefits for these lesions [7]. Therefore, it is recommended these lesions are simply observed if remaining asymptomatic and without growth, with MRI follow-ups and clinical observation for signs of hemorrhage [6, 7]. Surgical resection is indicated for RICMs presenting with symptomatic hemorrhage, recurrent hemorrhage, progressive neurological deficits, drug-resistant epilepsy (especially in the temporal lobe), and significant radiological progression indicating more aggressive behavior of the RICM [7, 14, 23]. In these cases, it has been demonstrated that surgery can markedly improve symptoms and prevent future hemorrhages and complete surgical resection if durable and often curative [32]. Microsurgical approaches are often preferred for accessible lesions, especially in the presence of symptomatic or recurrent hemorrhage [7]. However, the surgical benefits need to be balanced against the risk of neurological deficits, especially if the RICMs are in eloquent brain areas, and histopathological confirmation should be confirmed post-surgery [7]. There are also indications in the literature that incomplete resection can lead to rapid recurrence [6, 32]. Radiosurgery has also been postulated as a treatment modality, especially for surgically inaccessible lesions or aggressive lesions not amenable to conventional surgery [6]. A precise radiation dose delivery could help stabilize the lesion and reduce hemorrhage risk [6]. However, the long-term outcomes for pediatric RICMs are uncertain, and there are potential risks of further radiation-induced injury and post-radiosurgery hemorrhage [6]. Radiosurgery is therefore generally not recommended for accessible lesions [14]. Lastly, the utility of propranolol in the management of RICMs has been recently debated. Propranolol has shown promising results in the treatment of infantile hemangiomas, similar in some respects to cerebral cavernomas [33]. Its ability to induce ability to induce vasoconstriction, inhibit angiogenesis, and promote apoptosis could potentially be beneficial in reducing the size and impact of vascular lesions like cavernomas [33]. Propranolol could provide a non-invasive treatment alternative to surgery, especially for patients facing higher surgical risks [33]. However, the effectiveness of propranolol for cerebral cavernomas has been inconsistent in literature so far [33]. The optimal dosage is also unknown for cerebral cavernomas and the dose used for hemangiomas (2 mg/kg/day) may not be effective, and higher doses have not been thoroughly tested or approved for cerebral cavernomas [33]. Further larger-scale prospective studies are needed to explore this modality further.

Long-term prognostic data are largely missing from available literature, with no specific mentions of outcomes for 82.8% of our pooled patient cohort. It is hard to discern from the wording of available studies whether these patients have been followed up, remained asymptomatic, and are therefore not mentioned or whether the prognostic data were not collected at all. Of the available prognostic data, the reporting standard and follow-up periods vary widely. The fact that the majority of observed patients remained stable and the majority of surgical patients improved after surgery supports the management rationales described in the paragraph. However, more detailed reporting of long-term patient outcomes for both observation and surgical and radiosurgical interventions is needed to draw a better picture of patients’ quality of life with these lesions.

While our study pooled together the largest available patient cohort from known literature, it has several limitations. Only a single researcher was responsible for screening articles, and extracting and analyzing the data, leaving room for error, even when checks have been conducted to minimize this possibility. Furthermore, as we have implemented very few exclusion criteria based on article type, due to the nature of the published literature, this introduced high variability in the quality of synthesized data. As mentioned, the majority of the studies were case reports or small case series, introducing high bias risks and degrading the quality of the overall pooled data. The large variety in data reporting, resulting in many data categories including large amounts of “unknown” labels also reduced the overall confidence in the synthesized data. Further larger-scale prospective studies should be carried out in this field before further large-scale reviews to bolster confidence in the currently available literature findings.

## Conclusion

In conclusion, from 325 reviewed previously irradiated patients with subsequent RICM development, there appears to be a slight male predominance (3:2). RICMs appear to present about 10 years after the initial irradiation in late teen years and present incidentally in the majority of cases. There appears to be a predominance of RICMs in supratentorial locations. Asymptomatic RICMs are mostly observed over time, while lesions that have a predisposition to bleeding or demonstrate radiological progression are often operated on. Long-term data on prognostic outcomes for these patients are missing and larger-scale prospective studies with more detailed reporting are required to bolster the confidence in currently available literature findings and to shed more light on the unclear areas of RICM risk factors, management, and prognosis.

## Data Availability

No datasets were generated or analysed during the current study.

## Abbreviations

IPDA: Individual patient data analysis
RICM: Radiation-induced cavernous malformation
